# ‘Floc and Sink’ Technique Removes Cyanobacteria and Microcystins from Tropical Reservoir Water

**DOI:** 10.3390/toxins13060405

**Published:** 2021-06-08

**Authors:** Renan Silva Arruda, Natália Pessoa Noyma, Leonardo de Magalhães, Marcella Coelho Berjante Mesquita, Éryka Costa de Almeida, Ernani Pinto, Miquel Lürling, Marcelo Manzi Marinho

**Affiliations:** 1 Laboratory of Ecology and Physiology of Phytoplankton, Department of Plant Biology, University of Rio de Janeiro State, Rua São Francisco Xavier 524—PHLC Sala 511a, Rio de Janeiro 20550-900, Brazil; np.noyma@gmail.com (N.P.N.); demagalhaesleonardo@gmail.com (L.d.M.); macobeme@gmail.com (M.C.B.M.); manzi.uerj@gmail.com (M.M.M.); 2 Department of Clinical and Toxicological Analyses, School of Pharmaceutical Sciences, University of São Paulo, São Paulo 05508-900, Brazil; erykaca@usp.br (É.C.d.A.); ernani@usp.br (E.P.); 3 Aquatic Ecology & Water Quality Management Group, Department of Environmental Sciences, Wageningen University, P.O. Box 47, 6700 AA Wageningen, The Netherlands; miquel.lurling@wur.nl; 4 Department of Aquatic Ecology, Netherlands Institute of Ecology (NIOO-KNAW), P.O. Box 50, 6700 AB Wageningen, The Netherlands

**Keywords:** toxic bloom, cyanobacteria mitigation, geo-engineering, *Dolichospermum*, *Microcystis*, eutrophication control

## Abstract

Combining coagulants with ballast (natural soil or modified clay) to remove cyanobacteria from the water column is a promising tool to mitigate nuisance blooms. Nevertheless, the possible effects of this technique on different toxin-producing cyanobacteria species have not been thoroughly investigated. This laboratory study evaluated the potential effects of the “Floc and Sink” technique on releasing microcystins (MC) from the precipitated biomass. A combined treatment of polyaluminium chloride (PAC) with lanthanum modified bentonite (LMB) and/or local red soil (LRS) was applied to the bloom material (mainly *Dolichospermum circinalis* and *Microcystis aeruginosa*) of a tropical reservoir. Intra and extracellular MC and biomass removal were evaluated. PAC alone was not efficient to remove the biomass, while PAC + LMB + LRS was the most efficient and removed 4.3–7.5 times more biomass than other treatments. Intracellular MC concentrations ranged between 12 and 2.180 µg L^−1^ independent from the biomass. PAC treatment increased extracellular MC concentrations from 3.5 to 6 times. However, when combined with ballast, extracellular MC was up to 4.2 times lower in the top of the test tubes. Nevertheless, PAC + LRS and PAC + LMB + LRS treatments showed extracellular MC concentration eight times higher than controls in the bottom. Our results showed that Floc and Sink appears to be more promising in removing cyanobacteria and extracellular MC from the water column than a sole coagulant (PAC).

## 1. Introduction

Cyanobacteria perform many vital functions to the health of ecosystems, especially as photosynthetic organisms (e.g., oxygen production and nitrogen-fixing); however, they have become an increasing issue worldwide, mainly due to the massive proliferation of toxin-producing species caused by anthropogenic eutrophication [1,2,3]. Cyanobacterial blooms represent a threat to human health and aquatic biota, mainly due to the potential contamination by toxins [1,2,3].

Cyanotoxins comprise many compounds with wide variations in the chemical structure, causing effects in exposed invertebrates and vertebrates. In freshwaters, the most common cyanotoxin is the hepatotoxic MC, which presents more than 300 known variants [4]. MCs are cyclic heptapeptides with a general structure (Figure S1) composed of D-alanine, a variable amino acid (X1), D-MeAsp (D-erythro-β-methylaspartic acid), another variable amino acid (X2), Adda ((2S,3S,4E,6E,8S,9S)-3-amino-9-methoxy-2,6,8-trimethyl-10-phenyldeca-4,6-dienoic acid), D-glutamic acid, and Mdha (*N*-methyldehydroalanine). MCs have been named according to the standard one-letter amino acid code applied to the variable amino acids X1 and X2 (present in the positions 2 and 4, respectively). For example, the one-letter codes A, R, L, F, Y, and W present in some MCs variants (e.g., MC-RR, MC-LR, MC-LA, MC-LF, MC-LW, MC-YR, Figure S2, Table S1) represent the amino acids alanine (A), arginine (R), leucine (L), phenylalanine (F), tyrosine (Y), and tryptophan (W). Other structural modifications are added to the name as a suffix, such as [D-Asp3] which represents a missing methyl group in the position 3 of MCs structure (e.g., [D-Asp3]MC-RR and [D-Asp3]MC-LR, Figure S2) [3]. Studies have reported that MCs were shown to be tumor promoters, immunotoxicants, and endocrine disruptors [5,6,7]. This toxin can inhibit protein phosphatases 1 and 2A, promote DNA fragmentation, necrosis, apoptosis, and intrahepatic bleeding, leading to death [1,8]. MC-producing cyanobacteria blooms have been described in 80 countries, and the genera *Microcystis* and *Planktothrix* are considered the most potent sources because they are often associated with high toxin levels [9]. However, the filamentous genus *Dolichospermum* (formerly *Anabaena)* contains species capable of producing MCs [9] and are considered as potential MC producers in risk assessments [3], mainly strains from high latitudes [10,11,12]. MC-producing strains have also been recorded at moderate latitude [13] and low latitude [14,15].

Although the most coherent way to mitigate eutrophication is to reduce the external nutrient input [2,16], it is also an onerous approach in developing countries, where the sewage system and treatment are inefficient, requiring expensive investments [17]. Furthermore, there is a need to control the internal nutrient load to accelerate the system’s recovery [18].

The coagulation and precipitation of cyanobacteria biomass and phosphate (P) is a promising tool to manage eutrophication and its nuisance [19]. This technique combines a ballast and a coagulant (Floc and Sink) that moves intact cells and P out of the water column, bound to ballast, toward the sediment, and is proven safe [19,20,21,22,23]. However, the coagulation step is a critical part of this technique because the coagulant can cause physiological or chemical stress to cell membranes, releasing intracellular toxins and P into water [20,21,24]. Polyaluminium chloride (PAC) is widely used as an inorganic flocculant in drinking water supply plants [25,26], wastewater treatment [27], and in-lake restoration measures to mitigate cyanobacterial blooms [23,28,29,30]. Its mechanism of coagulation includes charge neutralization, sweep coagulation, and bridge aggregation [31]. Algal blooms coagulation can suffer interference from the release of algogenic organic matter (AOM) into the water, extracellularly (EOM), and intracellularly (IOM) when cell lysis occurs [32]. One mechanism AOM interferes in the coagulation efficiency is by forming complexes with cations [33]. More specifically, studies have shown that proteins from *Microcystis aeruginosa* interfere with coagulation by forming chelate complexes with the coagulant [34,35]. PAC also interacts with humic acid, reducing its effectiveness [36,37]. The reduction of humic acid is important in lake restoration measurements because of it interferes in phosphorus adsorption by lanthanum modified clay [38,39]. Although all these cited interferences can affect the flocculation efficiency, each water body is unique and has physical, chemical and biological characteristics. In each system, algal removal can be achieved by conducting experimental trials aiming to find the right flocculant and the best dosage [28,40,41]. In our study, PAC was chosen because it previously showed high efficiency to flocculate cyanobacterial blooms at the Funil reservoir [23,42]. In addition to PAC, LMB and LRS as a ballast present similar efficiency to settle the cyanobacteria with similar dosages [21,23,28]. Both compounds are solid-phase P sorbents (SPB), but also the removal of extracellular harmful algal toxins from the water has already been recorded for them [21,43]. Floc and Sink proved to be efficient in laboratory tests, cleaning the water column without indicating cell lysis [19]. Moreover, water from a tropical eutrophic reservoir could be cleared from cyanobacteria [23,42]. At the time of those studies, the samples comprised predominantly small spherical/flat colonial cyanobacteria (e.g., *Microcystis aeruginosa*, *Microcystis brasiliensis*, *Microcystis panniformis*). The majority of Floc and Sink studies have been performed with *Microcystis* [41,42,44]. Some studies, however, indicated that the efficiency of the Floc and Sink technique in cell removal varied among cyanobacteria species [21]. A recent study also mentioned that “the applicability of the technique to genera of cyanobacteria that have not yet been studied is unknown” [44]. One of the genera of which no information exists about how well it can be removed from water by the Floc and Sink technique is the filamentous genus *Dolichospermum* which recently became dominant in the phytoplankton community of a tropical eutrophic reservoir in the southeast region of Brazil (Funil Reservoir).

Since controlling eutrophication and mitigating cyanobacteria nuisance have been considered a crucial challenge to agencies and companies responsible for producing and distributing potable water [21,23], insight into the removal efficacy of filamentous *Dolichospermum* species is needed. The experiments executed here tested the hypothesis that the Floc and Sink technique efficiently removes the cyanobacterial biomass composed mainly of *Dolichospermum circinalis* with undergrowth of *Microcystis aeruginosa* in a deep tropical reservoir without MC releasing.

## 2. Results

### 2.1. Coagulant Range

**Figure 1:**
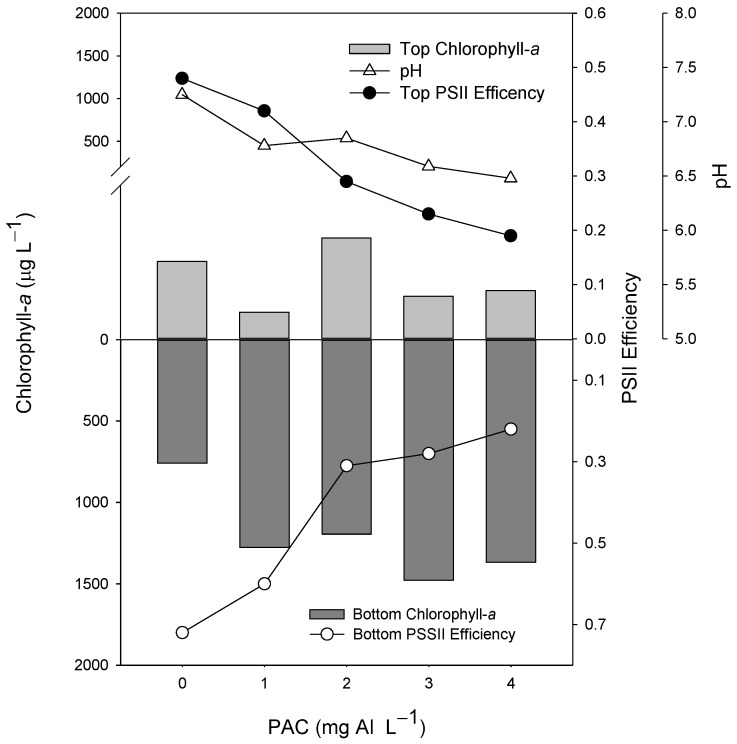
Chlorophyll-*a* concentrations (µg L^−1^) in the top 5 mL (top light gray bars) and bottom 5 mL (lower dark gray bars), Photosystem II efficiency (Φ_PSII_) (circles), and pH values (triangles) of 60 mL cyanobacteria suspensions incubated for 2 h in the presence of different concentrations (1, 2, 3, and 4 mg Al L^−1^) of the coagulant PAC (polyaluminium chloride). The control is represented by 0 mg Al L^−1^.

In the experiment testing different doses of PAC, the cyanobacteria suspension presented no positive buoyancy, and after the incubation (2 h), most of the biomass was in the bottom of the tube, as shown in the control (Figure 1). The chlorophyll-*a* concentration in the top 5 mL of the tubes containing PAC varied from 1.6 to 1.9 times less than in the bottom 5 mL of the control tube (Figure 1). The best dosage was 3 mg L^−1^, yielding the high biomass concentration in the bottom of the tube. The pH decreased gradually with the increasing dose of PAC concentrations, from 1 mg L^−1^ (Figure 1). Moreover, the Photosystem II efficiency (Φ_PSII_) reduced progressively in the top and bottom 5 mL in the PAC range tubes from 1 mg L^−1^.

### 2.2. Floc and Sink Assays

**Figure 2:**
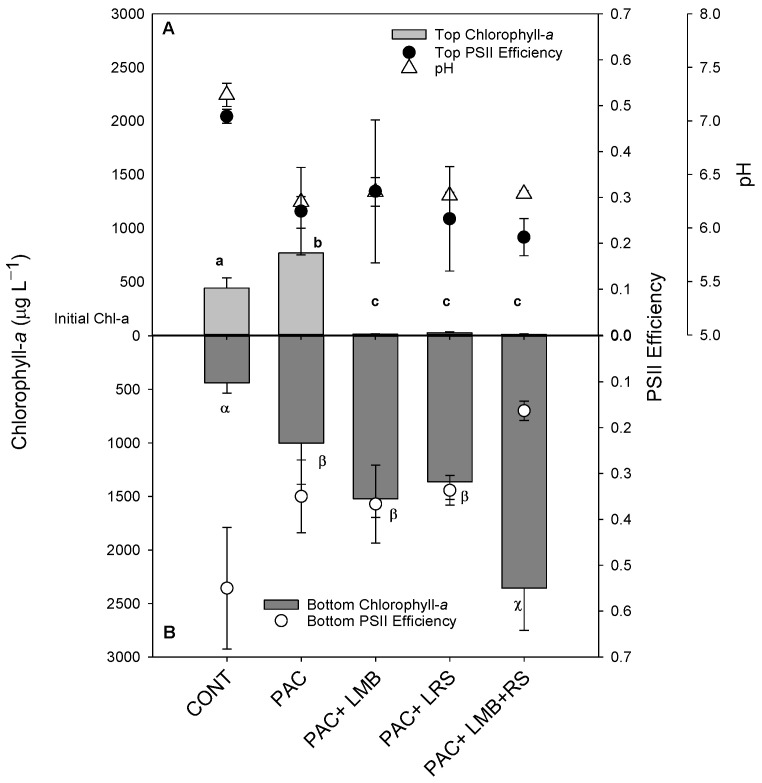
Chlorophyll-*a* concentrations (µg L^−1^) in the top 15 mL ((**A**) top light gray bars) and bottom 15 mL ((**B**) lower dark gray bars), Photosystem II efficiency (Φ_PSII_) in the top ((**A**) filled circles), and bottom ((**B**) open circles) and pH values ((**A**) triangles) of 1 L cyanobacteria suspensions from the Funil Reservoir incubated for 2 h in the absence (control) or presence of the coagulant (polyaluminium chloride, PAC 3 mg Al L^−^^1^) and coagulant combined with ballast (lanthanum modified bentonite, LMB 0.2 mg L^−1^, and local red soil, LRS 0.2 mg L^−1^) separately or in binary mixtures (lanthanum modified bentonite, LMB 0.1 mg L^−1^, and local red soil, LRS 0.1 mg L^−1^). The dotted line indicates the initial chlorophyll-*a* concentration in the cylinders, error bars represent one standard deviation *(n* = 3), and similar letters indicate homogeneous groups according to the Holm–Sidak post-hoc test (*p* < 0.05).

The cyanobacterial suspension in this experiment presented similar distribution in the water column. As indicated in the controls, the chlorophyll-*a* concentrations in the top of the cylinders were similar to the bottom, even after incubation (Figure 2). The addition of only PAC doubled the concentration of cyanobacterial biomass in both areas of the cylinders (Figure 2). This effect was strongly modified when PAC was combined with a ballast. Virtually all biomass was precipitated to the bottom of the cylinders with the addition of LMB and/or LRS (Figure 2). The chlorophyll-*a* concentration in the top of the cylinders in these treatments with ballast was 20 to 60 times lower than in the controls (Figure 2A), reaching a removal efficiency of 97 ± 1.8%, whereas in the bottom of the cylinders, there was 4.3 to 7.5 times more chlorophyll-*a* than in the control (Figure 2B). One-way ANOVA indicated a significant difference in chlorophyll-*a* concentrations among treatments for the top (F_4,14_ = 28.275; *p* < 0.001) and the bottom (F_4,14_ = 22.910; *p* < 0.001) of the cylinders. In the bottom of the cylinders, three homogeneous groups for chlorophyll-*a* concentrations were found: (i) the lowest concentration was observed in the control; (ii) similar to each other, but higher values than control were found for PAC, PAC + LMB, and PAC + LRS; (iii) the highest concentrations were detected in the PAC + LMB + LRS treatment (Figure 2B).

The Φ_PSII_ did not show a difference among treatments in the top of the cylinders (F_4,10_ = 3.259; *p* > 0.001), but Φ_PSII_ of PAC + LMB + LRS treatment was significantly lower than the control and other treatments in the bottom of the cylinders (*p* = 0.002) (Figure 2A,B). Differences were observed in pH between the control and all treatments (F_4,10_ = 79.083; *p* < 0.001) (Figure 2).

### 2.3. Effects on the Microcystins Concentrations

**Figure 3:**
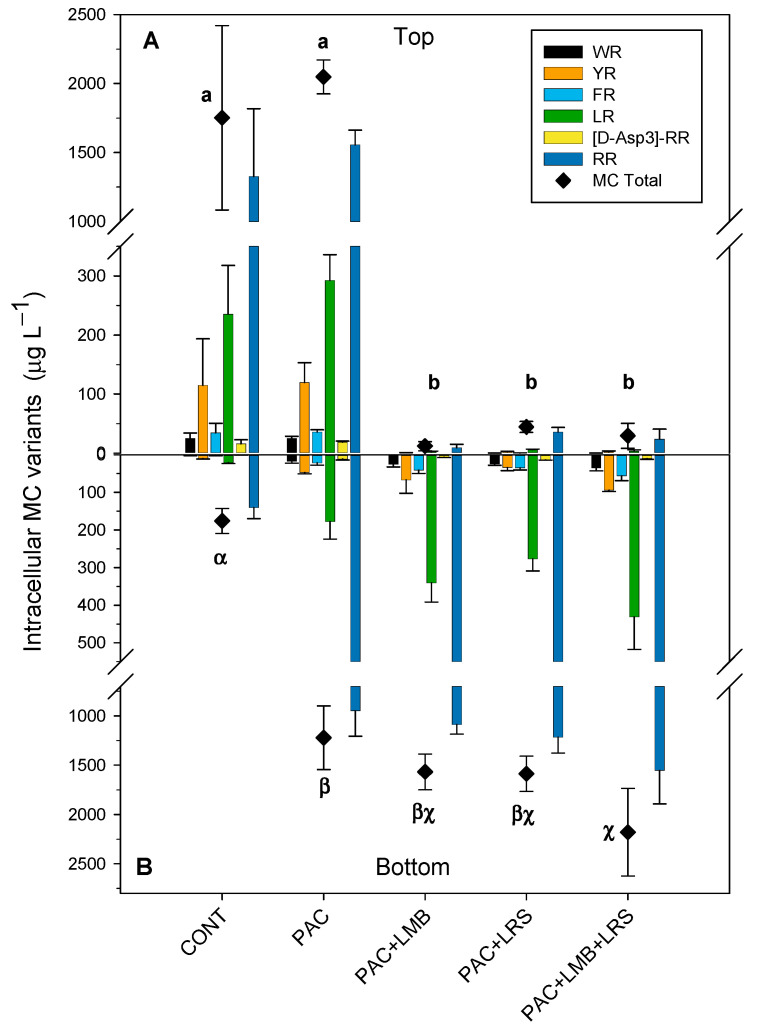
Intracellular concentrations (µg L^−1^) of MCs and their variants in the top of the cylinder (**A**) and bottom (**B**) in 1 L of cyanobacteria suspensions from the Funil Reservoir incubated for 2 h in the absence (control) or presence of the coagulant (polyaluminium chloride, PAC 3 mg Al L^−^^1^) and coagulant combined with ballast (lanthanum modified bentonite, LMB 0.2 mg L^−1^, and local red soil, LRS 0.2 mg L^−1^) separately or in binary mixtures (lanthanum modified bentonite, LMB 0.1 mg L^−1^, and local red soil, LRS 0.1 mg L^−1^). The black diamonds represent the total intracellular MC in the treatments. Similar letters indicate homogeneous groups in the total MC according to the Holm–Sidak post-hoc test (*p* < 0.05).

Total intracellular MC concentration in the top of the cylinders varied from 12 to 2049 µg L^−1^ (Figure 3A) and in the bottom, from 176 to 2180 µg L^−1^ (Figure 3B). Total intracellular MC values in the top of the cylinders (Figure 3A) were significantly higher in the control and the PAC treatment alone than in the other treatments (F_4,12_ = 59.980; *p* < 0.001). Total intracellular MC concentration in the top of the treatments combining PAC with a ballast was 40 times (PAC + LMB) to 148 times (PAC + LRS) lower than in the control. We also observed a significant difference between total intracellular MC concentrations in the bottom of the cylinders (F_4,13_ = 23.326; *p* < 0.001). The PAC + LMB + LRS treatment had the highest intracellular MC concentration in the bottom and was different from the control and PAC treatment (Figure 3B). No difference was recorded in the bottom of the tubes among treatments that combined PAC and a ballast (Figure 3B). Total intracellular MC concentration in treatments with a ballast was 7 to 12 times higher than in the control.

**Figure 4:**
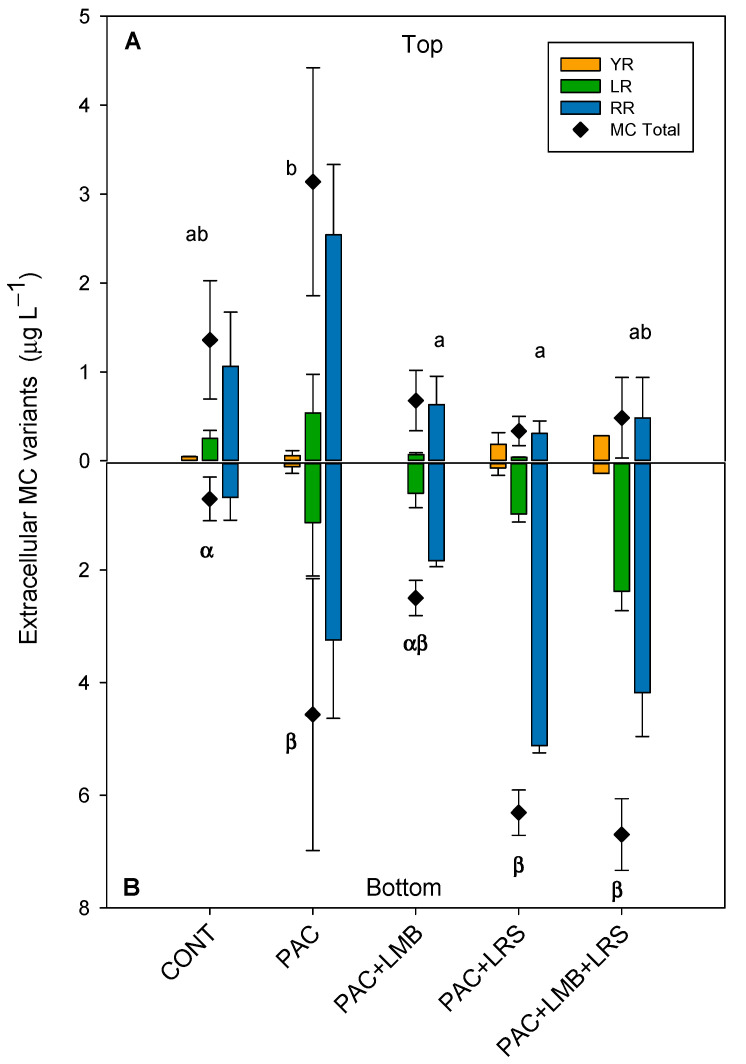
Extracellular concentrations (µg L^−1^) MCs and their variants in the top of the cylinder (**A**) and bottom (**B**) in 1 L of cyanobacteria suspensions from the Funil Reservoir incubated for 1 h in the absence (control) or presence of the coagulant (polyaluminium chloride, PAC 3 mg Al L^−1^) and coagulant combined with ballast (lanthanum modified bentonite, LMB 0.2 mg L^−1^, and local red soil, LRS 0.2 mg L^−1^) separately or in binary mixtures (lanthanum modified bentonite, LMB 0.1 mg L^−1^, and local red soil, LRS 0.1 mg L^−1^). The black diamonds represent the total intracellular MC in the treatments. Similar letters indicate homogeneous groups in the total MC according to the Holm–Sidak post-hoc test (*p* < 0.05).

Six MC variants were detected in the intracellular samples (WR, YR, FR, LR, and [D-Asp^3^] RR), while only three of those were found in extracellular samples (YR, LR, and RR). RR was the most abundant variant in both the intra- and extracellular samples, followed by LR and YR (Figure 3 and Figure 4). In samples with chlorophyll-*a* <11 µg L^−1^, the variants WR, FR, and [D-Asp^3^] RR were not detected. The concentration of the intracellular MC variants varied between treatments in the top and the bottom of the cylinders. The RR variant was the most abundant, representing an average of 69% in the top and 73% at the bottom of the cylinders, whereas the YR was less abundant, representing an average of 6% and 3% in the tube’s top and bottom, respectively.

Total extracellular MC concentrations in the top of the cylinders were considerably lower than intracellular concentrations and varied between 0.33 and 3.14 µg L^−1^ (Figure 4A). Although PAC increased 2.3 times the extracellular MC concentration, when combined with LMB, it reduced 75% of the MC concentration. PAC + LMB + LRS and PAC + LRS reduced 64% and 50%, respectively. One-way ANOVA indicated significant differences in total extracellular MC concentrations in the top of the cylinders (F_4,10_ = 8.174; *p* = 0.003). The PAC treatment was significantly different from the PAC + LMB and PAC + LRS and similar to PAC + LMB + LRS (Figure 4A). Nevertheless, no difference was recorded among treatments with ballast and control at the top (Figure 4A). The treatments combining PAC and ballast had 1 to 4.2 times less extracellular MC in the top than treatment with PAC only. Extracellular MC concentrations were also different among treatments in the bottom of the cylinders (F_4,6_ = 9.433; p = 0.009). The Holm–Sidak post-hoc test separated the extracellular MC concentrations in the bottom of the cylinders into two groups: (i) PAC, PAC + LRS, and PAC + LMB + LRS and (ii) control (Figure 4B). The highest concentration of extracellular MCs (5.67 µg L^−1^) in the bottom of the cylinders was observed in the PAC + LMB + LRS treatment (Figure 4B). The ANCOVA analysis showed no influence of biomass on the extracellular MC concentration in the top (F_1,9_ = 2.127; *p* = 0.179) and in the bottom (F_1,8_ = 1.534; *p* = 0.251).

## 3. Discussion

This study tested the hypothesis that the Floc and Sink technique could efficiently remove the cyanobacterial biomass comprised of filamentous *Dolichospermum* and small colonial *Microcystis* species without releasing MCs in water from a tropical reservoir. Our results indicated that biomass composed predominantly of *Dolichospermum circinalis* and *Microcystis aeruginosa* could be efficiently removed from the water column using a mixture of low-dose coagulants and ballasts. PAC in combination with LMB or LRS had similar efficacy, and this result is in agreement with previous studies carried out in tropical systems [21,22,23,24]. At odds with what was observed by Miranda et al. [21] and Lucena-Silva et al. [22], we recorded the release of MCs with a low dose of PAC (3 mg Al L^−1^) and in all combinations of PAC and ballast. The highest extracellular MC concentration was recorded in PAC + LMB + LRS, the most efficient treatment in removing biomass. Nevertheless, the decrease in MC concentration in the top of the cylinders treated with PAC + LMB and PAC + LRS and the lower concentration of MC compared to solely PAC suggests the clays’ potential capacity as ballast to remove dissolved MC from the water column [45,46,47].

### 3.1. Coagulant Range

Algal morphology and other characteristics (e.g., motility, surface charge, and extracellular organic matter) influence the coagulation and sedimentation processes [48]. In laboratory conditions, Miranda et al. [21] showed that similar concentrations of PAC resulted in distinct responses for blooms of the filamentous *Raphidiopsis raciborskii* (formerly *Cylindrospermopsis raciborskii*) and the colonial *Microcystis aeruginosa*. The filamentous species (*R. raciborskii*) formed flocs and subsequently sank, whereas the colonial species accumulated at the surface. Experiments with water samples dominated by the filamentous species *Planktothrix agardhii* and *R*. *raciborskii* also presented a tendency to sink in a wide range of PAC concentrations (2–32 mg Al L^−1^) [22]. In our experiment, the biomass concentration was higher at the bottom of the tube than at the top, possibly reflecting the coagulant effect on the dominant filamentous species, *Dolichospermum circinalis.* Based on these studies executed in freshwater, we could suggest a pattern where the filamentous species response to PAC is to sink, and colonial spherical tends to float. This hypothesis is also supported by differences between our results and the other study performed in the same system [23], in which the dominant species were the colonial spherical *Microcystis brasiliensis* (formerly *Sphaerocavum brasiliense*) and *Microcystis panniformis*. With a similar design, the experiment tested a range from 1 to 32 mg Al^−1^ of PAC, and in all concentrations, the biomass presented positive buoyance, accumulating in the top of the tubes [19].

We recorded a gradual decline in pH during the experiment, starting from 7 in control and 6.8 in the dose of 1 mg Al L^−1^ to 6.5 in the highest PAC dose of 4 mg L^−1^, values considered safe to the cyanobacteria’s physiological status [18]. Because hydrogen ions are released during hydrolysis of aluminum-based coagulants, they may cause a decline in the pH, promoting cell lysis [23,49]. There are reports of this phenomenon in the literature from doses ≥8 mg Al^−1^ [21,23], almost three times more than the 3 mg Al L^−1^ dose chosen as the optimal dose in this study. The Φ_PSII_ was not affected up to 1 mg of AL L^−1^ and decrease sharply from this concentration, being at odds with other studies where the decrease was recorded from 8 mg Al L^−1^ [41,50,51]. Miranda et al. [21] observed a reduction not only in the Φ_PSII_ but also in the pH in water dominated by *Microcystis* when using higher doses of PAC (16 and 32 mg Al L^−1^) in the PAC range test. These results suggest that there is no direct action of the PAC on the Φ_PSII_, which may vary according to the organism’s physiological state or vary between species.

### 3.2. Floc and Sink of Cyanobacteria

As we expected, the Floc and Sink experiment showed that *D. circinalis* and *M. aeruginosa* could be precipitated using a combination of a low dose of PAC and LMB and LRS as a ballast. These results agree with other studies conducted in tropical freshwater systems [19,52,53]. Several coagulants and clays (as ballast) have been tested, aiming to find a safe, efficient, and cheaper treatment [21,23,42]. In tropical aquatic ecosystems, both combinations (PAC + LMB and PAC + LRS) have shown similar efficacy in removing cyanobacterial biomass in laboratory tests [21,23]. The LMB is known as the commercial name Phoslock [54], is more expensive than LRS, a material easily found in banks of natural aquatic systems in the southeast of Brazil [51]. However, LMB has a great advantage over LRS: it immobilizes more P per unit product than LRS [51]. We also decided to test the efficacy of these two materials together (LMB + LRS) combined with PAC to reduce the cost of the potential application. All tested treatments had similar total ballast dosages. However, the treatment with PAC + LMB + LRS removed 38% more biomass than the treatment with just a single ballast (PAC + LMB or LRS).

The lanthanum of LMB has a strong affinity for P, forming an electrostatic interaction with flocks of biomass previously formed by charge neutralization of PAC [19,21,23,28]. This process turns these flocks heavy, sinking the cyanobacteria biomass. Natural soils present less affinity for P but can perform other mechanisms inducing sedimentation [23]. Previous tests in the Funil Reservoir using PAC + LRS and PAC + LMB showed excellent flocculation and sedimentation of biomass formed by *D. circinalis* and *M. aeruginosa* and was achieved without effects on the zeta potential [23]. On that occasion, the authors suggested inter-particle bridging and sweeping as a mechanism of flocculation followed by sedimentation. In other laboratory studies, the combined use of a coagulating metal salt and colloidal local soil solution has been shown to increase the electrostatic interaction, bridging, and enmeshment, which enhanced the effective collision between algal cells and clay particles and inducing sedimentation [44]. Considering this information, we suggest that the combined use of PAC + LMB + LRS were able to increase removal efficiency due to firstly, the action of the different chemical flocculation mechanisms provided by two different ballast, and secondly, the presence of inorganic particles with different granulometry, increasing the action of the physical mechanism in biomass flocks of different sizes, which led to improved sweep flocculation. It is noteworthy that the LRS is composed of coarse sand (2–0.0 mm), fine sand (0.20–0.05 mm), silt (0.05–0.002 mm), and clay (<0.002 mm) [23].

Surprisingly, a reduction in Φ_PSII_ was observed in the bottom of the cylinders when LMB and LRS were used together. This parameter expresses the Photosystem II efficiency, giving a more realistic evaluation of the physiological status of the cells under the tested conditions [23,55,56]. However, we cannot assume that a reduction of 30% in Φ_PSII_ reflects cell lysis [57]. A reduction in ΦPSII was already recorded using PAC (2 mg Al L^−1^) + ≤100 mg LMB L^−1^ in tests with the filamentous species *Planktothrix rubescens*, but there was no evidence of cell damage [19,50,58]. Miranda et al. [21] used 4 mg of Al L^−1^ in the filamentous species *R. raciborskii* and 8 mg of Al L^−1^ in the small colonial *Microcystis* in the Floc and Sink experiment and observed a marginal effect on the pH and Φ_PSII_, indicating no damaging of the cells during the incubation time. Thus, based on our data of pH and Φ_PSII,_ we cannot affirm the occurrence of the cell lysis, although it seems the stress in Φ_PSII_ may have stimulated MC liberation.

### 3.3. Effects of Floc and Sink Technique on the MCs Concentration

The Floc and Sink technique was proposed as a tool to manage eutrophication and cyanobacterial blooms [19,50,58]. One of the advantages of this technique is the possible coagulation and sinking of cells without lysis [19,50,58] and a later degradation of cyanobacteria and their toxins in the sediment [59,60]. In this way, we expected that the pH and Φ_PSII_ would remain steady or with low variations, not causing cell damage, and that the intra and extracellular MC amounts would remain unchanged after treatment.

In this study, six variants of MC were identified in the intracellular form. The less abundant variants could not be quantified in the low biomass. Only the three most abundant intracellular MC variants were also identified in the extracellular MC samples. Although in our experiments, the dosage of PAC (3 mg Al L^−1^) was below a critical dosage described in the literature, and the pH remained stable and under a safe level [19,21,22,23,49], we recorded variations in intra- and extracellular MC values among the control and the treatments. Firstly, we cannot generalize the physiological responses; different cyanobacteria species will respond differently to the same treatment, and different strains of the same species may also respond differently [57], so this precludes direct comparison with other studies. Second, we also cannot consider that a variation in the intracellular MC portion is just a consequence of the release. We have biomass comprised of a natural phytoplankton community, predominantly composed of two different species with morphological differences and probably various strains producing MC or not. In that view, different species or strains may possess different buoyancy, as exemplified in the controls by the discrepancy between the biomass (chlorophyll-*a*) and the measured intracellular MC concentration. Whereas chlorophyll-*a* concentrations in both the top and the bottom of the tubes were similar (see Figure 2), intracellular MC concentrations in the top were 10 times higher than in the bottom (see Figure 3A,B). In addition, we have the influence of the treatment under all these variables mentioned above. An evaluation of the coagulation properties of ten microalgae and cyanobacteria species showed that not just the type of coagulant and dosage influence the effectiveness of the coagulation process but also the species [61]. Based on this, we presume that species characteristics (e.g., morphology, mucilage, surface charge, etc.) could affect the coagulation process. Thus, it was not expected that the distribution of species and, consequently, the intracellular MC of flocculated biomass in the cylinders occurs homogeneously. In this way, with the methodology adopted in this study, we did not consider any variation in the intracellular MC portion as an irrefutable indicator of toxin release.

Among the coagulants commonly used to remove cyanobacterial biomass, PAC is safer than other aluminum-based salt coagulants because a lower dose is needed to obtain efficient results. Consequently, there is less pH reduction, preventing cell lysis during the settling process and the liberation of intracellular toxins [62,63]. However, we observed an increase of 2.3 and 4.6 times in the extracellular MC in the bottom and top of the cylinders, respectively, for PAC treatment compared to control. Unlike our findings, Miranda et al. [21] applied a dose of 4 mg Al L^−1^ in water samples dominated by *R. raciborskii* and *Microcystis* spp. and had not observed any significant alteration in saxitoxins or MC concentration. Lucena et al. [22] reported that PAC had not affected the extracellular MC fraction, indicating no cell lysis in experiments using water samples dominated by *P. agardhii* and *R. raciborskii*. An explanation for the divergences between our results and those in the published literature would be the initial pH; here it was ~7 while the similar tests were around 8–10 [21,22]. Another explanation is that the tests already carried out were comprised of biomass of different cyanobacteria species. There are no data about the coagulation process with *Dolichospermum* species. Another possibility is the physiological stage of cells in our samples. The release of toxins appears to occur mainly, but not exclusively, during cellular senescence [3]. From a physiological perspective, the PAC solution can be more toxic when the cells are in the senescence phase [3].

In the Floc and Sink experiment, we sampled both the top and bottom of the cylinders. We observed that the extracellular MC concentration was higher in the bottom of the cylinders, giving the impression that it was higher in more dense biomasses. Although the covariance analysis (ANCOVA) has shown that there is no effect of biomass on extracellular MC, we cannot ignore that the cell lysis may not happen immediately after the application of the treatment, occurring after cells sedimentation [24] and promoting more extracellular MC concentration in more dense biomasses. It may also happen during the filtration process due to initial slight cell damage promoted by PAC added to the mechanical action of the sampling process. Furthermore, MC release may not occur due to cell lysis specifically but because of the chemical stress response. Coagulation tests with polyaluminium ferric chloride and the filamentous species *R. raciborskii* reported increasing extracellular saxitoxins even without any indicator of cell lysis [21]. The authors attributed this result to the fact that filamentous species are more susceptible to external stress than small colonial species. In this study, we did not use any direct test of cell lysis. However, considering the incubation time and our results, we can say that lysis did not occur immediately after applying of the technique but after the sinking of the cyanobacteria.

Additionally, MCs’ bioavailability in the water column is influenced by suspended particles’ adsorption, especially clays [45,64]. The MC desorption process was already described, and the stability of this bound MC depends on the clays’ composition [65,66]. Hence, MC molecules, which are unstably connected to ballast particles after sedimentation, were released by adverse reactions, increasing extracellular MC levels in the bottom of the cylinders. Nonetheless, more research is needed to disentangle possible coagulant MC-liberating and ballast MC-adsorbing/desorbing effects. Some studies have indicated that a Floc and Sink treatment can reduce biomass and MCs strongly [57], while LMB itself could lower extracellular MC concentration by 61–86% [46].

There are some possibilities for the non-homogeneity in dissolved MC in the water column, first when the tests are carried out with a natural phytoplankton population and large water volumes. Another reason is the potential property of clays in reducing the concentration of dissolved MC. LMB and LRS adsorption may have indirectly influenced dissolved MC concentration. The PAC + LMB treatment showed a significant reduction in extracellular MC (73%) compared to only PAC treatment on the top of the cylinders. If PAC promoted MC release, as observed by comparing to control, the reduction recorded in PAC + LMB treatment could be due to LMB sorption. These findings agree with the recently reported capacity of LMB to lower extracellular MC concentration [45,64]. A similar activity could be observed in PAC + LRS treatment. The extracellular MC decreased by 88% in the top of the cylinders. Laboratory studies showed that sediment particles applied in the water column effectively reduced MC concentration to less than the detection limit [45,64]. Noteworthy, no significant difference was observed in the bottom of the cylinders in all treatments.

Unexpectedly, no difference was recorded in MC-extra in the top of the cylinders between PAC + LMB + LRS and PAC treatment. A possible explanation for the divergence between this treatment and others with ballast is that the ballast compound showed no linear adsorption capacity. Investigating the ability to absorb MC by LMB, the authors observed that at the doses of 50, 100, and 150 ppm, the decrease in MC concentrations was 61.2%, 86.0%, and 75.4%, respectively, relative to the control [50,60]. Many studies have focused on the adsorptive capacity of MC in sediment [50,60], but little is known about the potential of MC adsorption by lanthanum modified bentonite (LMB). These facts would justify the differences observed in extracellular MC concentration between treatments that contained ballast since we think that MCs’ release was promoted by adding PAC in all ballast treatments.

We emphasize that in this study, at the top of the cylinders in all treatments combining coagulant and ballast, the extracellular MC concentration was below 1 µg L^−1^, the maximum acceptable values adopted by WHO [3] and the Brazilian Government. Although we observed an increase in extracellular MC concentration after application of the Floc and Sink technique, especially in the bottom of the cylinders of PAC, PAC + LRS, and PAC + LMB + LRS treatments, these extracellular MC amounts are negligible compared with the intracellular concentrations that range from 1.2 to 2.2 µg L^−1^ in the same treatments. For instance, in the most efficient treatment in removing biomass, PAC + LMB + LRS, it is less than 0.3%. Moreover, some authors consider that lysis of cyanobacteria after sedimentation of biomass is beneficial in reducing the possibility of recolonization of the water column by perturbations or bioturbation process, whereas near the sediment, liberated toxins can be adsorbed and degraded by decomposing bacteria [50,60]. These results show that Floc and Sink is a promising tool to manage toxic blooms. However, we suggest a detailed investigation of PAC effects on different species of toxin-producing cyanobacteria, and additionally, an investigation of the potential capacity for MCs adsorption in LMB and clays is needed.

## 4. Conclusions

Our results showed that combining a low dose of coagulant with a ballast compound effectively removed biomass composed predominantly of *Dolichospermum circinalis* and *Microcystis aeruginosa* from the water column. PAC + LMB + LRS was the most efficient combination to settle the cyanobacteria biomass in the bottom of the tubes. PAC promoted the release of MC but combined with ballast, and the concentrations remained similar to control in the top. PAC + LMB was efficient to remove biomass and maintained concentrations of extracellular MC similar to the control. Although we observed an increase in extracellular concentrations of MC in the bottom of the tube after the application of PAC + LRS and LMB + LRS, the concentrations were extremely low, which does not preclude technical use.

## 5. Materials and Methods

### 5.1. Sampling

The Funil Reservoir is a eutrophic system located in southern Brazil (22°30′ S and 44°45′ W) at 440 m of altitude, in a warm-rainy tropical climate area (Cwa in the Köppen system). Since the 1980s, toxigenic cyanobacterial blooms with a dominance of *Microcystis aeruginosa* have been registered in this reservoir, especially during warm periods [67,68].

For the experiments, water samples were collected in the mild-cold season and concentrated with a plankton net (50 µm mesh) to create a cyanobacterial suspension, yielding an initial concentration of ~155 µg L^−1^ of chlorophyll-*a*. The total cyanobacteria biomass in the sample was composed of 72% *Dolichospermum circinalis* (Rabenhorst) Wacklin, Hoffmann and Komárek, 23% *Microcystis* *aeruginosa* (Kützing) Kützing, 3% *Dolichospermum spiroides* (Klebhan) Wacklin, L. Hoffmann and Komárek, and 1% *Microcystis panniformis* Komárek et al. At the time of sampling, the pH was 7.38, alkalinity was 499 µEq L^−1^, turbidity was 16 NTU, and the water temperature was 21 °C.

### 5.2. Chemicals and Materials

The coagulant PAC (polyaluminium chloride; Al_n_(OH)_m_Cl3_n—m_, r ~1.37 kg L^−1^, 9.5% Al, 21.0% Cl) was obtained from Purewater Efluentes (São Paulo, Brazil). Local red soil (LRS) was collected from the banks of the Funil Reservoir as described by Noyma et al. (2016), and the lanthanum modified bentonite Phoslock^®^ (LMB) was obtained from HydroScience (Porto Alegre, Brazil). LRS and LMB were used as ballast.

### 5.3. Floc and Sink Assays

#### 5.3.1. Experiment 1—Coagulant Range

A range of PAC concentrations were tested (0, 1, 2, 3, and 4 mg Al L^−1^). This experiment has no replicates because it followed a regression design to evaluate the most appropriate and effective coagulant dosage. The assay was set up in glass tubes (10 × 200 mm) containing 60 mL of cyanobacteria suspensions with an initial concentration of ~155 µg L^−1^ of chlorophyll-*a*. PAC was added at the surface and mixed with a metal rod for 30 s; the tubes were then incubated for two hours. After the incubation time, the tubes were visually inspected for flocs formation, and 5 mL aliquots were taken from the top and bottom of the tubes to determine the precipitation of cyanobacteria biomass. The chlorophyll-*a* concentration and the Photosystem II efficiency (Φ_PSII_) [55] were measured using a PHYTO-PAM phytoplankton analyzer (Heinz Walz GmbH, Effeltrich, Germany). The pH was also measured in the tubes.

#### 5.3.2. Experiment 2—Floc and Sink Assays

Based on the first experiment, the coagulant (PAC) dose of 3 mg of Al L^−1^ was chosen as an effective dose. This 3 mg Al L^−1^ of PAC was also combined with LMB (0.2 g L^−1^), LRS (0.2 g L^−1^), and LMB + LRS (0.1 g^−1^ of each). The dosage of ballast was based on previous tests [21,23]. The experiment was run in triplicate in acrylic cylinders containing 1 L of cyanobacteria suspensions from the Funil Reservoir. We tested four treatments: (1) only PAC, (2) PAC + LMB, (3) PAC + LRS, and (4) PAC + LMB + LRS, while the fifth series remained untreated (Controls). The PAC was added first, followed by the immediate addition of a slurry of LMB and/or LRS in the top of the cylinders. Subsequently, the suspensions were mixed with a glass stirring rod, and after two hours, 15 mL samples were collected from the top and bottom of the cylinders. Then, 8 mL of sample were filtered through 1.2 µm glass fiber filters (85/70 BF, Macherey-Nagel) to quantify MCs. The filters were used to quantify intracellular MC, and the filtrate was used to quantify extracellular MC. Filter and filtrate samples were immediately frozen and kept at −20 °C until the analysis. Then, 5 mL of the samples were used to quantify chlorophyll-*a* and Φ_PSII_ by PHYTO-PAM phytoplankton analyzer (Heinz Walz GmbH, Effeltrich, Germany).

### 5.4. Sample Analysis

#### Toxins Analysis

Before the extractions, filters, and filtrates were freeze-dried (Sartorius GmbH, Germany), for the analysis of the intracellular toxins, 2.5 mL of 75% (*v*/*v*) methanol (MeOH) (Merck^®^, São Paulo, Brazil) was added to the filters in an 8 mL glass tube. After vortexing the samples for 15 s, they were placed in a water bath (Buchi Heating Bath b-491, São Paulo, Brazil) for 30 min at 60 °C. The suspensions were transferred to new glass tubes. This extraction step was repeated twice, but this time with 2.0 mL of 75% (*v*/*v*) methanol. The new glass tubes containing 6.5 mL of extract were placed in the Speedvac (Savant SPD121P, Thermo Scientific, Waltham, MA, USA). After drying, they were resuspended with 2 mL of MeOH 100% (*v*/*v*), vortexed for 15 s and filtered through 0.45 μm PVDF membrane syringe filters (Analítica, São Paulo, Brazil) into amber glass vials for LC-MS/MS analysis. For the extracellular toxins analysis, the freeze-dried samples were resuspended with 2 mL of MeOH 100% (*v*/*v*), vortexed for 15 s, and filtered through 0.45 μm PVDF membrane syringe filters (Analítica, Brazil) into amber glass vials. If needed, the samples with high MC concentrations were diluted in methanol 75% *v*/*v* before re-analysis. The lyophilized samples were resuspended with 2 mL of MeOH (100%), vortexed for 15 s, and filtered through 0.45 μm PVDF membrane syringe filters (Analítica, Brazil) into amber vials for the analysis of dissolved toxins. Samples were then stored in a freezer (−20 °C) until LC-MS/MS analysis. See the Supplementary Materials for additional details on MC recovery (Table S2).

Toxin determination in all samples was performed by liquid chromatography-tandem mass spectrometry (LC-MS/MS) using a series 200 HPLC system (Perkin Elmer, Waltham, MA, USA) coupled to electrospray ionization (ESI) mass spectrometer. Chromatographic separations were carried out on a Luna C18 column (150 × 2 mm; 5 μm particles, Phenomenex, Torrance, CA, USA). The mobile phases consisted of 5 mM ammonium formate and 53 mM formic acid in water (mobile phase A) and 90% (*v*/*v*) acetonitrile (mobile phase B). Gradient elution was performed at a flow rate of 300 μL min^−1^ and followed a linear increase from 10 to 90% B within 15 min, then it was held at 90% B for 2 min, and returned to the initial condition (10% B) within 12 min. MS/MS experiments were performed using an API 365 triple quadrupole (QqQ) mass spectrometer (AB Sciex, Concord, ON, Canada) equipped with a turbo ion spray source. The instrument was operated using the selected reaction monitoring (SRM) mode, with specific *m/z* transitions selected for the highest sensitivity and selectivity. Single and double-charged ions were monitored in positive ion mode. Characteristic precursor ions for SRM were *m/z* 519 (RR), 910 (LA), 986 (LF), 995 (LR), 1025 (LW), 1045 (YR), 512 ([D-Asp^3^] RR), and 981 ([D-Asp^3^] LR). As only MCs were detected in the studied samples, MCs’ quantification in SRM mode was based on the characteristic Adda fragment at *m/z* 135. Calibration standards of non-demethylated MCs were obtained from Abraxis (Eurofins^®^, Nantes, France) and prepared in methanol 75%. Samples were quantified using a calibration curve and subsequently corrected for recovery. Quantification of the demethylated MC structures was performed by relative quantification using the corresponding non-demethylated MC as analytical standards.

### 5.5. Statistical Analysis

A one-way ANOVA analysis was performed to evaluate the differences in chlorophyll-*a* and MC concentration between treatments in the tool pack SigmaPlot (12.5 version, Rio de Janeiro, Brazil). A pairwise multiple comparison post-hoc test (Holm–Sidak) was applied to distinguish means that were significantly different (*p* < 0.05). The ANCOVA analysis was also performed in IBM SPSS Statistics^®^ (2.0 version, Rio de Janeiro, Brazil) to evaluate the linear relationship between biomass and MC concentration.

## Supplementary Materials

The following are available online at https://www.mdpi.com/article/10.3390/toxins13060405/s1, Figure S1: Microcystins (MCs) general structure, cyclo-(*D*-Ala^1^-X_1_^2^-*D*-MeAsp3-X_2_^4^-Adda^5^-*D*-Glu^6^-Mdha^7^). *D*-Ala: D-alanine, X_1_ and X_2_: variable amino acids, *D*-MeAsp: *D*-erythro-β-methylaspartic acid, Adda: (2S,3S,4E,6E,8S,9S)-3-amino-9-methoxy-2,6,8-trimethyl-10-phenyldeca-4,6-dienoic acid, D-Glu: D-glutamic acid, Mdha: *N*-methyldehydroalanine. R_1_ and R_2_: H or CH_3_., Figure S2: Chemical structure for the MC variants MC-RR, [*D*-Asp3]MC-RR, MC-YR, MC-LR, [*D*-Asp3]MC-LR, MC-LA, MC-LF, and MC-LW. The one-letter codes A, R, L, F, Y, and W represent the amino acids alanine, arginine, leucine, phenylalanine, tyrosine, and tryptophan, respectively. The suffix [*D*-Asp3] represents a missing methyl group in position 3 of MCs structure. Table S1: Chemical and physical properties of the MC variants MC-RR, [*D*-Asp3]MC-RR, MC-YR, MC-LR, [*D*-Asp3]MC-LR, MC-LA, MC-LF, and MC-LW., Table S2: Recovery in % for MC in two different matrices (tap water and cultures of Chlorella vulgaris) for three concentrations.

## References

[1] Chorus I., Bertram J., World Health Organization (1999). Toxic Cyanobacteria in Water: A Guide to Their Public Health Consequences, Monitoring, and Management.

[2] Huisman J., Codd G.A., Paerl H.W., Ibelings B.W., Verspagen J.M.H., Visser P.M. (2018). Cyanobacterial Blooms. Nat. Rev. Microbiol..

[3] Chorus I., Welker M., World Health Organization (2021). Toxic Cyanobacteria in Water: A Guide to Their Public Health Consequences, Monitoring and Management.

[4] Jones M.R., Pinto E., Torres M.A., Dörr F., Mazur-Marzec H., Szubert K., Tartaglione L., Dell’Aversano C., Miles C.O., Beach D.G. (2020). Comprehensive Database of Secondary Metabolites from Cyanobacteria. Water Res..

[5] Lankoff A., Carmichael W.W., Grasman K.A., Yuan M. (2004). The Uptake Kinetics and Immunotoxic Effects of Microcystin-LR in Human and Chicken Peripheral Blood Lymphocytes in Vitro. Toxicology.

[6] Nishiwaki-Matsushima R., Ohta T., Nishiwaki S., Suganuma M., Kohyama K., Ishikawa T., Carmichael W.W., Fujiki H. (1992). Liver Tumor Promotion by the Cyanobacterial Cyclic Peptide Toxin Microcystin-LR. J. Cancer Res. Clin. Oncol..

[7] Rojas M., Nuñez M.T., Zambrano F. (1990). Inhibitory Effect of a Toxic Peptide Isolated from a Waterbloom of Microcystis sp. (Cyanobacteria) on Iron Uptake by Rabbit Reticulocytes. Toxicon.

[8] Dziga D., Maksylewicz A., Maroszek M., Budzyńska A., Napiorkowska-Krzebietke A., Toporowska M., Grabowska M., Kozak A., Rosińska J., Meriluoto J. (2017). The Biodegradation of Microcystins in Temperate Freshwater Bodies with Previous Cyanobacterial History. Ecotoxicol. Environ. Saf..

[9] Li X., Dreher T.W., Li R. (2016). An Overview of Diversity, Occurrence, Genetics and Toxin Production of Bloom-Forming Dolichospermum (Anabaena) Species. Harmful Algae.

[10] Harada K.I., Ogawa K., Kimura Y., Murata H., Suzuki M., Thorn P.M., Evans W.R., Carmichael W.W. (1991). Microcystins from Anabaena Flos-Aquae NRC 525-17. Chem. Res. Toxicol..

[11] Rantala A., Rajaniemi-Wacklin P., Lyra C., Lepistö L., Rintala J., Mankiewicz-Boczek J., Sivonen K. (2006). Detection of Microcystin-Producing Cyanobacteria in Finnish Lakes with Genus-Specific Microcystin Synthetase Gene E (McyE) PCR and Associations with Environmental Factors. Appl. Environ. Microbiol..

[12] Kobos J., Błaszczyk A., Hohlfeld N., Toruńska-Sitarz A., Krakowiak A., Hebel A., Sutryk K., Grabowska M., Toporowska M., Kokociński M. (2013). Cyanobacteria and Cyanotoxins in Polish Freshwater Bodies. Oceanol. Hydrobiol. Stud..

[13] Dreher T.W., Collart L.P., Mueller R.S., Halsey K.H., Bildfell R.J., Schreder P., Sobhakumari A., Ferry R. (2019). Anabaena/Dolichospermum as the Source of Lethal Microcystin Levels Responsible for a Large Cattle Toxicosis Event. Toxicon X.

[14] Sá L.L.C.D., Vieira J.M.D.S., Mendes R.D.A., Pinheiro S.C.C., Vale E.R., Alves F.A.D.S., Jesus I.M.D., Santos E.C.D.O., Costa V.B.D. (2010). Ocorrência de Uma Floração de Cianobactérias Tóxicas Na Margem Direita Do Rio Tapajós, No Município de Santarém (Pará, Brasil). Rev. Pan-Amaz. Saúde.

[15] Sant’Anna C.L., Azevedo M.T.D.P. (2000). Contribution to the Knowledge of Potentially Toxic Cyanobacteria from Brazil. Nova Hedwig..

[16] Paerl H.W., Gardner W.S., Havens K.E., Joyner A.R., McCarthy M.J., Newell S.E., Qin B., Scott J.T. (2016). Mitigating Cyanobacterial Harmful Algal Blooms in Aquatic Ecosystems Impacted by Climate Change and Anthropogenic Nutrients. Harmful Algae.

[17] van Loosdrecht M.C.M., Brdjanovic D. (2014). Anticipating the next Century of Wastewater Treatment. Science.

[18] Cooke G.D., Welch E.B., Peterson S., Nichols S.A. (2016). Restoration and Management of Lakes and Reservoirs.

[19] Lürling M., Kang L., Mucci M., van Oosterhout F., Noyma N.P., Miranda M., Huszar V.L.M., Waajen G., Marinho M.M. (2020). Coagulation and Precipitation of Cyanobacterial Blooms. Ecol. Eng..

[20] Liu H., Du Y., Wang X., Sun L. (2004). Chitosan Kills Bacteria through Cell Membrane Damage. Int. J. Food Microbiol..

[21] Miranda M., Noyma N., Pacheco F.S., de Magalhães L., Pinto E., Santos S., Soares M.F.A., Huszar V.L., Lürling M., Marinho M.M. (2017). The Efficiency of Combined Coagulant and Ballast to Remove Harmful Cyanobacterial Blooms in a Tropical Shallow System. Harmful Algae.

[22] de Lucena-Silva D., Molozzi J., dos Santos Severiano J., Becker V., de Lucena Barbosa J.E. (2019). Removal Efficiency of Phosphorus, Cyanobacteria and Cyanotoxins by the “Flock & Sink” Mitigation Technique in Semi-Arid Eutrophic Waters. Water Res..

[23] Noyma N.P., de Magalhães L., Furtado L.L., Mucci M., van Oosterhout F., Huszar V.L.M., Marinho M.M., Lürling M. (2016). Controlling Cyanobacterial Blooms through Effective Flocculation and Sedimentation with Combined Use of Flocculants and Phosphorus Adsorbing Natural Soil and Modified Clay. Water Res..

[24] Sun F., Pei H.Y., Hu W.R., Ma C.X. (2012). The Lysis of Microcystis Aeruginosa in AlCl 3 Coagulation and Sedimentation Processes. Chem. Eng. J..

[25] Zouboulis A., Traskas G., Samaras P. (2008). Comparison of Efficiency between Poly-Aluminium Chloride and Aluminium Sulphate Coagulants during Full-Scale Experiments in a Drinking Water Treatment Plant. Sep. Sci. Technol..

[26] Zarchi I., Friedler E., Rebhun M. (2013). Polyaluminium Chloride as an Alternative to Alum for the Direct Filtration of Drinking Water. Environ. Technol..

[27] Delgado S., Diaz F., Garcia D., Otero N. (2003). Behaviour of Inorganic Coagulants in Secondary Effluents from a Conventional Wastewater Treatment Plant. Filtr. Sep..

[28] Lürling M., Oosterhout F. (2013). van Controlling Eutrophication by Combined Bloom Precipitation and Sediment Phosphorus Inactivation. Water Res..

[29] Araújo F., dos Santos H.R., Becker V., Attayde J.L. (2018). The Use of Polyaluminium Chloride as a Restoration Measure to Improve Water Quality in Tropical Shallow Lakes. Acta Limnol. Bras..

[30] Kasprzak P., Gonsiorczyk T., Grossart H.P., Hupfer M., Koschel R., Petzoldt T., Wauer G. (2018). Restoration of a Eutrophic Hard-Water Lake by Applying an Optimised Dosage of Poly-Aluminium Chloride (PAC). Limnologica.

[31] Wei N., Zhang Z., Liu D., Wu Y., Wang J., Wang Q. (2015). Coagulation Behavior of Polyaluminum Chloride: Effects of PH and Coagulant Dosage. Chin. J. Chem. Eng..

[32] Her N., Amy G., Park H.R., Song M. (2004). Characterizing Algogenic Organic Matter (AOM) and Evaluating Associated NF Membrane Fouling. Water Res..

[33] Bernhardt H., Hoyer O., Lusse B., Schell H. Investigations on the Influence of Algal-Derived Organic Substances on Flocculation and Filtration. Proceedings of the National Conference on Drinking water: Treatment of Drinking Water for Organic Contaminants.

[34] Takaara T., Sano D., Konno H., Omura T. (2007). Cellular Proteins of Microcystis Aeruginosa Inhibiting Coagulation with Polyaluminum Chloride. Water Res..

[35] Takaara T., Sano D., Konno H., Omura T. (2004). Affinity Isolation of Algal Organic Matters Able to Form Complex with Aluminium Coagulant. Water Supply.

[36] Zhang P., Ren B.Z., Wang F. (2013). Humic Acid Removal from Water by Poly Aluminium Chloride (PAC). Appl. Mech. Mater..

[37] Sudoh R., Islam M.S., Sazawa K., Okazaki T., Hata N., Taguchi S., Kuramitz H. (2015). Removal of Dissolved Humic Acid from Water by Coagulation Method Using Polyaluminum Chloride (PAC) with Calcium Carbonate as Neutralizer and Coagulant Aid. J. Environ. Chem. Eng..

[38] Lürling M., Waajen G., van Oosterhout F. (2014). Humic Substances Interfere with Phosphate Removal by Lanthanum Modified Clay in Controlling Eutrophication. Water Res..

[39] Reitzel K., Balslev K.A., Jensen H.S. (2017). The Influence of Lake Water Alkalinity and Humic Substances on Particle Dispersion and Lanthanum Desorption from a Lanthanum Modified Bentonite. Water Res..

[40] Mucci M., Noyma N.P., de Magalhães L., Miranda M., van Oosterhout F., Guedes I.A., Huszar V.L.M., Marinho M.M., Lürling M. (2017). Chitosan as Coagulant on Cyanobacteria in Lake Restoration Management May Cause Rapid Cell Lysis. Water Res..

[41] de Magalhães L., Noyma N.P., Furtado L.L., Mucci M., van Oosterhout F., Huszar V.L.M., Marinho M.M., Lürling M. (2017). Efficacy of Coagulants and Ballast Compounds in Removal of Cyanobacteria (Microcystis) from Water of the Tropical Lagoon Jacarepaguá (Rio de Janeiro, Brazil). Estuaries Coasts.

[42] Noyma N.P., de Magalhães L., Miranda M., Mucci M., van Oosterhout F., Huszar V.L.M., Marinho M.M., Lima E.R.A., Lurling M. (2017). Coagulant plus Ballast Technique Provides a Rapid Mitigation of Cyanobacterial Nuisance. PLoS ONE.

[43] Pierce R.H., Henry M.S., Higham C.J., Blum P., Sengco M.R., Anderson D.M. (2004). Removal of Harmful Algal Cells (Karenia Brevis) and Toxins from Seawater Culture by Clay Flocculation. Harmful Algae.

[44] Thongdam S., Kuster A.C., Huser B.J., Kuster A.T. (2021). Low Dose Coagulant and Local Soil Ballast Effectively Remove Cyanobacteria (Microcystis) from Tropical Lake Water without Cell Damage. Water.

[45] Chen W., Song L., Peng L., Wan N., Zhang X., Gan N. (2008). Reduction in Microcystin Concentrations in Large and Shallow Lakes: Water and Sediment-Interface Contributions. Water Res..

[46] Dail H., Iv L., Lefler F., Berthold D.E. (2020). Sorption of Dissolved Microcystin Using Lanthanum-Modified Bentonite Clay. J. Aquat. Plant Manag..

[47] Prochazka E., Hawker D., Hwang G.S., Shaw G., Stewart I., Wickramasinghe W. The Removal of Microcystins in Drinking Water by Clay Minerals. Proceedings of the 14th International Conference on Harmful Algae.

[48] Li H., Pei H., Xu H., Jin Y., Sun J. (2018). Behavior of Cylindrospermopsis Raciborskii during Coagulation and Sludge Storage-Higher Potential Risk of Toxin Release than Microcystis Aeruginosa?. J. Hazard. Mater..

[49] Han J., Jeon B.S., Park H.D. (2012). Cyanobacteria Cell Damage and Cyanotoxin Release in Response to Alum Treatment. Water Sci. Technol. Water Supply.

[50] Pan G., Zou H., Chen H., Yuan X. (2006). Removal of Harmful Cyanobacterial Blooms in Taihu Lake Using Local Soils. III. Factors Affecting the Removal Efficiency and an in Situ Field Experiment Using Chitosan-Modified Local Soils. Environ. Pollut..

[51] Mucci M., Maliaka V., Noyma N.P., Marinho M.M., Lürling M. (2018). Assessment of Possible Solid-Phase Phosphate Sorbents to Mitigate Eutrophication: Influence of PH and Anoxia. Sci. Total. Environ..

[52] Lürling M., Faassen E.J. (2013). Dog Poisonings Associated with a Microcystis Aeruginosa Bloom in the Netherlands. Toxins.

[53] Pan G., Dai L., Li L., He L., Li H., Bi L., Gulati R.D. (2012). Reducing the Recruitment of Sedimented Algae and Nutrient Release into the Overlying Water Using Modified Soil/Sand Flocculation-Capping in Eutrophic Lakes. Environ. Sci. Technol..

[54] Valsami-Jones E., International Water Association (2004). Phosphorus in Environmental Technology: Principles and Applications.

[55] Genty B., Briantais J.M., Baker N.R. (1989). The Relationship between the Quantum Yield of Photosynthetic Electron Transport and Quenching of Chlorophyll Fluorescence. Biochim. Biophys. Acta Gen. Subj..

[56] Weenink E.F.J., Luimstra V.M., Schuurmans J.M., van Herk M.J., Visser P.M., Matthijs H.C.P. (2015). Combatting Cyanobacteria with Hydrogen Peroxide: A Laboratory Study on the Consequences for Phytoplankton Community and Diversity. Front. Microbiol..

[57] Lürling M., Mucci M., Waajen G. (2020). Removal of Positively Buoyant Planktothrix Rubescens in Lake Restoration. Toxins.

[58] Faassen E.J., Lürling M. (2013). Occurrence of the Microcystins MC-LW and MC-LF in Dutch Surface Waters and Their Contribution to Total Microcystin Toxicity. Mar. Drugs.

[59] Grützmacher G., Wessel G., Klitzke S., Chorus I. (2010). Microcystin Elimination during Sediment Contact. Environ. Sci. Technol..

[60] Holst T., Jørgensen N.O.G., Jørgensen C., Johansen A. (2003). Degradation of Microcystin in Sediments at Oxic and Anoxic, Denitrifying Conditions. Water Res..

[61] Lama S., Muylaert K., Karki T.B., Foubert I., Henderson R.K., Vandamme D. (2016). Flocculation Properties of Several Microalgae and a Cyanobacterium Species during Ferric Chloride, Chitosan and Alkaline Flocculation. Bioresour. Technol..

[62] de Julio M., Fioravante D.A., de Julio T.S., Oroski F.I., Graham N.J.D. (2010). A Methodology for Optimising the Removal of Cyanobacteria Cells from a Brazilian Eutrophic Water. Braz. J. Chem. Eng..

[63] Gebbie P. Using Polyaluminium Coagulants in Water Treatment. Proceedings of the 64th Annual Water industry Engineers and Operators’ Conference.

[64] Chen X., Yang X., Yang L., Xiao B., Wu X., Wang J., Wan H. (2010). An Effective Pathway for the Removal of Microcystin LR via Anoxic Biodegradation in Lake Sediments. Water Res..

[65] Miller M.J., Hutson J., Fallowfield H.J. (2005). The Adsorption of Cyanobacterial Hepatoxins as a Function of Soil Properties. J. Water Health.

[66] Miller M.J., Critchley M.M., Hutson J., Fallowfield H.J. (2001). The Adsorption of Cyanobacterial Hepatotoxins from Water onto Soil during Batch Experiments. Water Res..

[67] Rangel L.M., Silva L.H.S., Rosa P., Roland F., Huszar V.L.M. (2012). Phytoplankton Biomass Is Mainly Controlled by Hydrology and Phosphorus Concentrations in Tropical Hydroelectric Reservoirs. Hydrobiologia.

[68] Soares M.C.S., Maria M.I., Marinho M.M., Azevedo S.M.F.O., Branco C.W.C., Huszar V.L.M. (2009). Changes in Species Composition during Annual Cyanobacterial Dominance in a Tropical Reservoir: Physical Factors, Nutrients and Grazing Effects. Aquat. Microb. Ecol..

