# Supplementary material for: ‘Floc and Sink’ Technique Removes Cyanobacteria and Microcystins from Tropical Reservoir Water
Source: Toxins (Basel). 2021 Jun 8;13(6):405. doi: 10.3390/toxins13060405 (PMC8228476; doi:10.3390/toxins13060405)
Supplement: Supplementary file 1 [file toxins-13-00405-s001.zip › toxins-1200840-supplementary.pdf]

Supplementary Materials: 'Floc and Sink' Technique Removes Cyanobacteria and Microcystins from Tropical Reservoir Water

Renan Silva Arruda, Natália Pessoa Noyma, Leonardo de Magalhães, Marcella Coelho Berjante Mesquita, Éryka Costa de Almeida, Ernani Pinto, Miquel Lürling and Marcelo Manzi Marinho

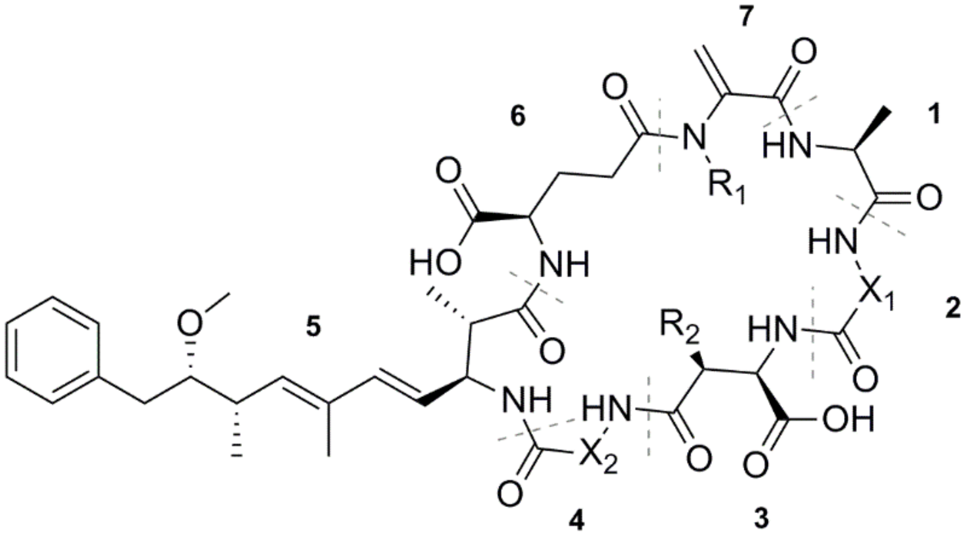

Figure S1. Microcystins (MCs) general structure, cyclo-(D-Ala^1-X_1^2-D-MeAsp3-X_2^4-Adda^5-D-Glu^6-Mdha^7). D-Ala: D-alanine, X_1 and X_2: variable aminoacids, D-MeAsp: D-erythro-β-methylaspartic acid, Adda: (2S,3S,4E,6E,8S,9S)-3-amino-9-methoxy-2,6,8-trimethyl-10-phenyldeca-4,6-dienoic acid, D-Glu: D-glutamic acid, Mdha: *N*-methyldehydroalanine. R_1 and R_2: H or CH_3.

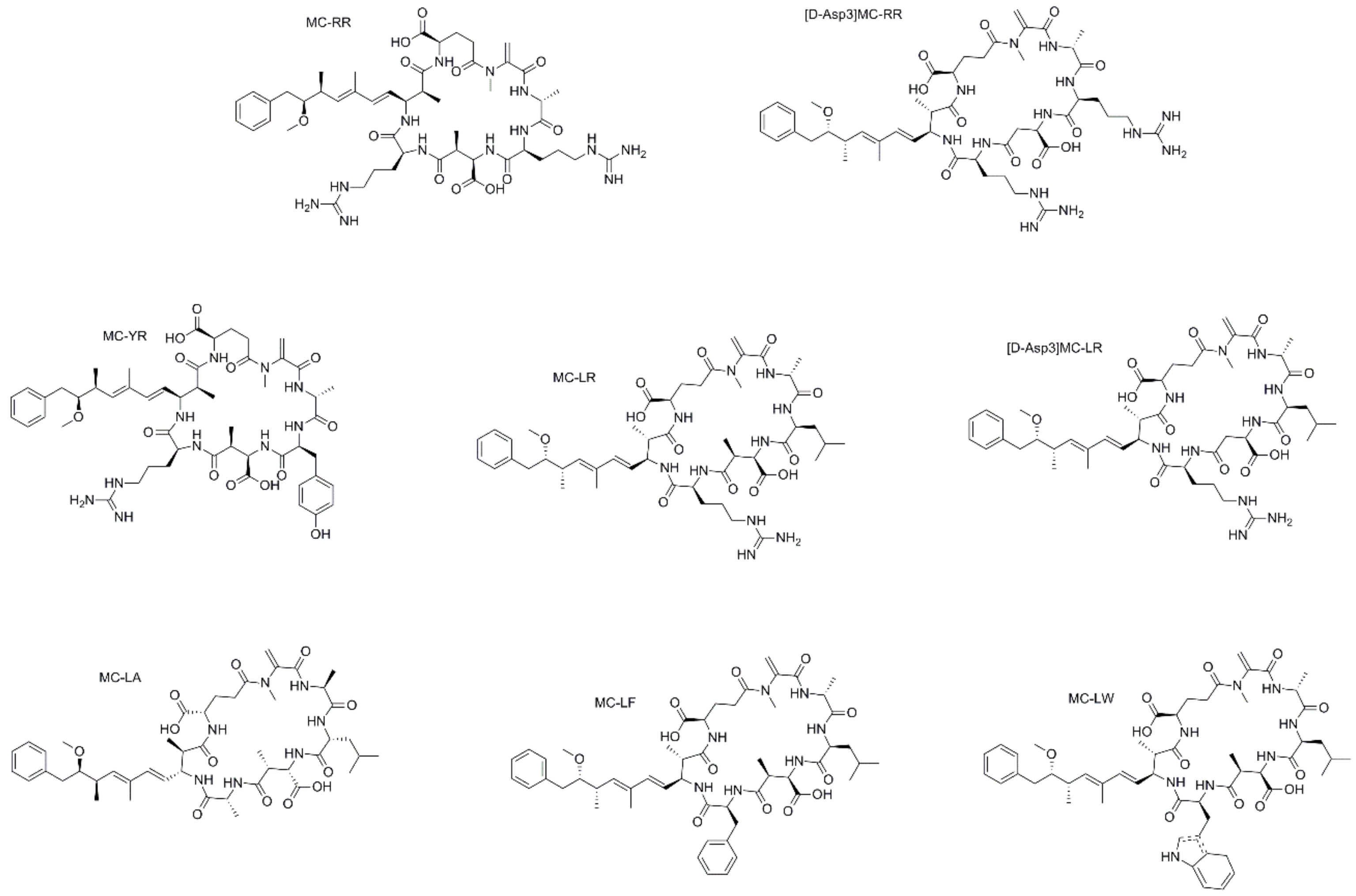

Figure S2. Chemical structure for the microcystin (MC) variants MC-RR, [D-Asp3]MC-RR, MC-YR, MC-LR, [D-Asp3]MC-LR, MC-LA, MC-LF, and MC-LW. The one-letter codes A, R, L, F, Y, and W represent the amino acids alanine, arginine, leucine, phenylalanine, tyrosine, and tryptophan. The suffix [D-Asp3] represents a missing methyl group in the position 3 of MCs structure.

Table S1. Chemical and physical properties of the microcystin (MC) variants MC-RR, [D-Asp3]MC-RR, MC-YR, MC-LR, [D-Asp3]MC-LR, MC-LA, MC-LF, and MC-LW.

Variants	Molecular Formula	MW (g mol^{-1})	CLogP *	pKa *
MC-RR	$C_{49}H_{75}N_{13}O_{12}$	1038.2	−2.44	2.94, 3.68
[D-Asp3]MC-RR	$C_{48}H_{73}N_{13}O_{12}$	1024.2	−2.96	2.93, 3.68
MC-YR	$C_{52}H_{72}N_{10}O_{13}$	1045.2	−0.02	2.89, 3.68, 9.40
MC-LR	$C_{49}H_{74}N_{10}O_{12}$	995.2	0.67	2.09; 2.19; 12.48 [a]
[D-Asp3]MC-LR	$C_{48}H_{72}N_{10}O_{12}$	981.1	0.16	2.93, 3.68
MC-LA	$C_{46}H_{67}N_{7}O_{12}$	910.1	4.61	2.98, 3.68
MC-LF	$C_{52}H_{71}N_{7}O_{12}$	986.2	6.03	2.91, 3.68
MC-LW	$C_{54}H_{72}N_{8}O_{12}$	1025.2	-	-

* Calculated by the ChemDraw 15.0 version. [a] Source: Gert-Jan de Maagd P et al; Wat Res 33: 677–80 (1999): https://www.ru.nl/publish/pages/533334/demaagdetal.1998a.pdf

Recovery was previously estimated in our in-house method for microcystins determination by LC-MS/MS, based on Bortoli et al, 2014 method (Growth and microcystin production of a Brazilian *Microcystis aeruginosa* strain (LTPNA 02) under different nutrient conditions. Brazilian Journal of Pharmacognosy, *24*, 4, 389–398. https://doi.org/10.1016/j.bjp.2014.07.019) and two PhD thesis (links are listed at the end of Table S2).

Briefly, the recovery of microcystins is the following: The study of recovery was performed by spiking the samples of either tap water (to simulate real samples of water) or powder of *Chlorella vulgaris* (to simulate cyanobacteria matrix) with standard solutions within the concentrations range of microcystins (10, 250 and 1000 μg L^{-1}). The recovery was estimated dividing values found for each microcystins "spiked" in the two matrices and the concentration values obtained with solutions of microcystins dissolved in the mobile phase. The recovery values ranged from 95.7 to 102 for matrix of tap water and 90.3 to 103.1 for Chlorella. Experiments were carried out in triplicate and represented as average and relative standard deviation (RSD). MC-LF, MC-LW, [D-Asp3]MC-RR and [D-Asp3]MC-LR were not calculated because of the lack of analytical standard to reach these concentrations.

Table S2. Recovery in % for microcystins in two different matrices (tap water and cultures of Chlorella vulgaris) for three concentrations.

Microcystin Variants	Concentration (Spiked) (μg L^{-1})	Recovery—Tap Water as Matrix	RSD (%)	Recovery—*Chlorella vulgaris* as Matrix	RSD (%)
MC-LR	10	98.3	2.1	103.1	2.3
	250	96.9	2.0	95.6	2.4
	1000	101.2	1.5	98.5	1.9
MC-YR	10	96.1	1.3	95.9	1.8
	250	98.0	1.2	97.2	2.1
	1000	93.8	1.7	95.2	2.9
MC-RR	10	95.7	1.0	99.3	1.3
	250	96.6	0.9	98.4	1.5
	1000	102.0	0.6	99.5	1.0
MC-LA	10	99.5	0.9	95.6	2.5
	250	94.6	1.6	93.5	2.4
	1000	91.5	2.1	90.3	3.5

These results were previously described in two PhD thesis supervised by Dr. Ernani Pinto (co-author of this manuscript): Kazumi Kinoshita—https://www.teses.usp.br/teses/disponiveis/9/9141/tde-23102015-144426/pt-br.php, Fabiane Dörr—https://www.teses.usp.br/teses/disponiveis/9/9141/tde-10062015-171941/pt-br.php.
